# Resource Utilization and Timeliness of Care in Patients With Incidentally Detected Lung Nodules

**DOI:** 10.1016/j.chpulm.2026.100270

**Published:** 2026-07-03

**Authors:** Kevin N. Zhou, Mina Habib, In-Lu Amy Liu, Michael K. Gould

**Affiliations:** a Kaiser Permanente Bernard J. Tyson School of Medicine, Kaiser Permanente Southern California, Pasadena, CA; b Department of Research and Evaluation, Kaiser Permanente Southern California, Pasadena, CA

**Keywords:** incidental lung nodules, lung cancer, Neighborhood Deprivation Index, resource utilization, socioeconomic disparities, timeliness of care

## Abstract

**Background:**

Management of patients with incidental lung nodules varies widely, leading to possible disparities in patient outcomes. Here, we describe resource utilization and timeliness of care for patients with incidental nodules measuring ≥ 9 mm in size, a threshold at which multiple options exist for evaluation. We further investigated the relationship between timeliness of care and socioeconomic context by using the Neighborhood Deprivation Index.

**Research Question:**

How do resource utilization and timeliness of care vary for patients with incidental lung nodules ≥ 9 mm, and how are these factors influenced by neighborhood socioeconomic deprivation?

**Study Design and Methods:**

A previously validated natural language processing algorithm identified 23,789 patients within a large, vertically integrated health system (2006-2016) with incidental lung nodules ≥ 9 mm identified by chest CT scan. Utilization of imaging and invasive procedures was described, stratified by final diagnosis, and represented with Sankey plots. Timeliness of care was assessed using Kaplan-Meier curves and multivariable regression, with the Neighborhood Deprivation Index evaluated as both a categorical and continuous variable.

**Results:**

Among all patients with incidentally detected nodules, 2,356 were diagnosed with lung cancer (9.9%). Overall, follow-up imaging was obtained in 60.1% of patients with benign nodules and 92.9% of patients with malignant nodules. One in 5 surgical resections, 1 in 2 percutaneous biopsies, and 2 in 3 bronchoscopies were performed in patients with benign nodules, consistent with possible overevaluation. Sankey plots revealed more complex and variable care pathways for patients with malignant nodules. A 1-SD increase in deprivation was associated with an 11% reduction in the timeliness of treatment (hazard ratio, 0.89; 95% CI, 0.81-0.97), but there was no association with time to diagnosis.

**Interpretation:**

Our results show a wide variation in resource utilization and care pathways for patients with benign and malignant lung nodules. Socioeconomic deprivation was associated with increased time to cancer treatment.

**Take-Home Points**

**Study Question:** How do resource utilization and timeliness of care vary for patients with incidental lung nodules ≥ 9 mm, and how are these factors influenced by neighborhood socioeconomic deprivation?

**Results:** Among 23,789 patients with incidentally detected nodules ≥ 9 mm, 36.7% had no documented follow-up imaging. One in 5 surgical resections, 1 in 2 biopsies, and 2 in 3 bronchoscopies were performed in patients who ultimately did not have cancer. Although neighborhood deprivation was not associated with time to diagnosis, it was associated with delayed treatment.

**Interpretation:** Care pathways for patients with benign and malignant nodules varied widely, with evidence of both underevaluation and overevaluation, and socioeconomic deprivation was associated with a delay in cancer treatment.

Every year, approximately 1.6 million lung nodules are detected with chest CT scan.^1^^,^^2^ Clinical practice guidelines for lung nodule management, including guidelines from the American College of Chest Physicians (CHEST),^3^^,^^4^ typically provide weak recommendations based on low certainty evidence. For nodules measuring > 8 mm, management options include periodic CT scan surveillance, nonsurgical biopsy, functional imaging with PET scan, and primary surgical diagnosis, with decisions based on cancer risk, procedural risk, and patient factors.

Prior work within the Veterans Health Administration demonstrated that with variation in pulmonary nodule evaluation,^5^ patients received markedly different management strategies, including no follow-up or prolonged surveillance. This heterogeneity may impose substantial physical and economic burdens; however, generalizability was limited by the small, predominantly veteran population studied.

Zheng et al^6^ used natural language processing to identify > 70,000 pulmonary nodules among 23,789 patients using chest CT scan reports in a large, integrated health system. Data from this patient population have shown approximately 10% of patients with an incidental pulmonary nodule ≥ 9 mm in size will receive a lung cancer diagnosis.^2^^,^^7^ In this study, we leveraged this large cohort to extend the findings of the Veterans Health Administration study and provide novel information about nodule evaluation practices and the timeliness of care for patients with large incidental lung nodules.

This study focused on nodules ≥ 9 mm, a threshold at which multiple options for nodule evaluation exist. Accordingly, we described variation in management strategies and resource utilization for patients with incidentally detected pulmonary nodules measuring ≥ 9 mm and examined time to diagnosis and treatment for patients with malignant nodules. We further investigated the relationship using the Neighborhood Deprivation Index (NDI), a validated measure of socioeconomic status.^8^^,^^9^

## Study Design and Methods

This was a retrospective, observational study describing resource utilization and time to diagnosis and treatment among patients with lung nodules measuring 9 to 30 mm. Patients were Kaiser Permanente Southern California members who underwent chest CT scan between 2006 and 2016. Patients with nodules and nodule characteristics were identified using a validated natural language processing algorithm^6^ that scanned the free text of dictated radiology reports. Data were obtained from Kaiser Permanente sources, including the Research Data Warehouse^10^ (patient characteristics, resource utilization, and vital status), the Radiology Information System (nodule characteristics), and a local cancer registry (cancer incidence and treatment dates). NDI data were available for patients after 2012. Kaiser Permanente Southern California institutional review board approval was obtained (No. 5804).

Nodules were identified using natural language processing of radiology reports regardless of whether patients were participating in a formal lung cancer screening program. Because the study period (2006-2016) largely preceded widespread implementation of low-dose CT scan screening, screening status was not used as a stratification variable.

### Patients

The study included 23,789 individuals who had a natural language processing-identified nodule on the report of a chest CT scan performed for any indication during the period January 1, 2006, to December 31, 2016, as described previously.^8^ The sample was restricted to Kaiser Permanente members with 13 months of continuous membership after nodule identification, allowing a 31-day gap, to ensure outcome ascertainment. Patients with lung or extrapulmonary cancer diagnoses within 5 years of nodule identification were excluded (except nonmelanoma skin cancer), as such nodules carry a significantly increased pretest probability of malignancy and are often managed with different diagnostic assumptions.

### Exposures and Outcomes

To describe resource utilization after lung nodule identification, we reported statistics on radiologic surveillance (CT scan or chest radiograph), PET scan or PET-CT scan, lung biopsy (percutaneous or bronchoscopic), pleural procedures, extrathoracic biopsy, and thoracic surgical procedures, including mediastinoscopy/mediastinotomy and thoracoscopy/thoracotomy. We stratified the sample by nodule etiology (lung cancer vs other) to describe anticipated differences in management for patients with malignant and benign nodules. Resource use was tallied over 27 months for patients with benign nodules, but was truncated after the date of diagnosis for patients who were diagnosed with lung cancer.

To describe the timeliness of diagnosis and treatment for patients who had a lung cancer diagnosis within 27 months of nodule identification, and to examine the effect of neighborhood deprivation (exposure) on timeliness (outcome), we calculated the number of days from nodule identification to cancer diagnosis and the number of days from nodule identification to first treatment for patients with a malignant nodule. Treatment was defined as any chemotherapy, radiation, and surgical intervention, in addition to immunotherapies and targeted therapies (labeled systemic therapies).

Neighborhood-level deprivation was quantified using a validated index derived from American Community Survey census tract data.^8^^,^^9^ Principal component analysis was used to create this index based on the following variables: percent of the adult population with less than a high school diploma, percent of households earning < $30,000 per year, percent of households with below poverty-level income, proportion of civilian noninstitutionalized population between 18 and 64 years of age who are unemployed, proportion of households on public assistance, percent in crowded housing, proportion of households headed by female individuals (no male present) with dependent children, and percent of male individuals in management or professional occupations.

### Statistical Analysis

Resource evaluation metrics reported for each diagnostic test included the number and proportion of patients with at least 1 test and the total number of tests performed. Continuous variables were presented as mean ± SD and median (interquartile range). Categorical variables were presented as count (%). Sankey plots were generated to illustrate the various care pathways for patient management, with each round representing the next imaging test or diagnostic procedure for an individual patient after the index CT scan. Time to diagnosis and first treatment were measured in days. We reported median times to diagnosis and treatment (interquartile range) and plotted these timeliness measures using Kaplan-Meier curves.

To examine the association between the NDI and timeliness, the sample of patients with malignant nodules was stratified into NDI quartiles from least to most deprived. A score of 1 represented a 1-SD increase in deprivation from the mean. In the prespecified analytical plan, the NDI was analyzed categorically by quartile, with the least deprived quartile serving as the reference category. Post hoc, we also analyzed the NDI as a continuous variable. Plausible confounders were entered in multivariable models for adjustment purposes and included nodule characteristics (size, attenuation, and edge), patient characteristics (race, age, sex, insurance type, and smoking status), comorbidities (smoking history, BMI, Charlson Comorbidity Index, COPD, congestive heart failure, and renal disease), and medical center where care was received. Because all patients included in the time-to-diagnosis analysis experienced the event of interest within the 27-month follow-up period, there was no censoring, and time to diagnosis was therefore modeled using multiple linear regression. Patients with missing covariate data (< 6% for all covariates except smoking status, which was missing in 16% of all patients) were excluded from relevant models. The proportion of missingness varied by variable and is reported in Table 1. Time to first treatment was modeled using the Fine-Gray subdistribution hazard model to account for the censoring of patients who did not receive treatment and the competing risk of death. All statistical analyses were conducted in SAS, version 9.4 (SAS Institute Inc). The threshold for statistical significance was a 2-sided *P* < .05.

**Table 1:** Clinical and Demographic Characteristics

Characteristic	No Lung Cancer (n = 21,433)		Lung Cancer (n = 2,356)		Total (N = 23,789)
Age, mean [SD], y	64.5 [15.4]		69.8 [10.5]		65.0 [15.0]
Biological sex					
Female	11,348 (52.9)	89.8	1,290 (54.8)	10.2	12,638 (53.1)
Male	10,085 (47.1)	90.4	1,066 (45.2)	9.6	11,151 (46.9)
Race/ethnicity					
Asian	2,162 (10.7)	89.9	243 (10.6)	10.1	2,405 (10.7)
American Indian/Alaska Native	62 (0.3)	86.1	10 (0.4)	13.9	72 (0.3)
Black	2,407 (12)	89	299 (13.1)	11	2,706 (12.1)
Native Hawaiian/Pacific Islander	180 (0.9)	90.5	19 (0.8)	9.5	199 (0.9)
White	14,813 (73.6)	89.8	1,675 (73.3)	10.2	16,488 (73.6)
Other	489 (2.4)	92.8	38 (1.7)	7.2	527 (2.4)
Missing	1,320		72		1,392
Ethnicity					
Hispanic	4,673 (22.3)	93.8	310 (13.3)	6.2	4,983 (21.4)
Not Hispanic	16,266 (77.7)	89	2,020 (86.7)	11	18,286 (78.6)
Unknown or missing	494		26		520
Marital status					
Never married	2,895 (14.2)	93.8	191 (8.3)	6.2	3,086 (13.6)
Married or living as married	11,484 (56.3)	89.6	1,333 (58)	10.4	12,817 (56.5)
Widowed	3,552 (17.4)	88.7	452 (19.7)	11.3	4,004 (17.7)
Separated	290 (1.4)	92.9	22 (1)	7.1	312 (1.4)
Divorced	2,149 (10.5)	87.9	297 (12.9)	12.1	2,446 (10.8)
Other	10 (0)	83.3	2 (0.1)	16.7	12 (0.1)
Missing	1,053		59		1,112
Education					
< 9th grade	1,962 (9.8)	90.5	205 (9.1)	9.5	2,167 (9.7)
9-12th grade	371 (1.8)	88.8	47 (2.1)	11.2	418 (1.9)
High school graduate	5,432 (27.1)	90	603 (26.7)	10	6,035 (27)
Some college, no degree	7,263 (36.2)	89.7	830 (36.7)	10.3	8,093 (36.2)
Associate degree	10 (0)	90.9	1 (0)	9.1	11 (0)
Bachelor degree	46,18 (23)	89.8	525 (23.2)	10.2	5,143 (23)
Graduate degree	409 (2)	88.9	51 (2.3)	11.1	460 (2.1)
Missing	1,368		94		1,462
Household median income					
No.	20,061		2,261		22,322
Mean [SD]	65,873.6 [29,165.5]		65,489.3 [27,996.8]		65,834.7 [29,048.9]
Missing	1,372		95		1,467
Smoking status					
Currently smokes	2,124 (11.8)	82.3	457 (23.3)	17.7	2,581 (13)
Formerly smoked	7,351 (41)	87.8	1,025 (52.2)	12.2	8,376 (42.1)
Never (or passively) smoked	8,473 (47.2)	94.6	481 (24.5)	5.4	8,954 (45)
Unknown or missing	3,485		393		3,878
Pack-years (among patients who currently smoke/formerly smoked)					
No.	5,207		916		6,123
Mean [SD]	28.5 [26.8]		39.7 [36.1]		30.2 [28.7]
Missing	4,268		566		4,834
Quit years (among patients who formerly smoked)					
No.	6,034		849		6,883
Mean [SD]	21.9 [16.26]		17.1 [14.54]		21.3 [16.13]
Missing	1,317		176		1,493
BMI, kg/m^2^					
No.	20,042		2,175		22,217
Mean [SD]	27.6 [6.8]		26.7 [6.0]		27.5 [6.8]
Missing	1,391		181		1,572
Charlson Comorbidity Index score					
No.	21,417		2,355		23,772
Mean [SD]	1.7 [1.8]		1.7 [1.7]		1.7 [1.8]
Missing	16		1		17
Charlson Comorbidity Index comorbidities					
Myocardial infarction	2,004 (9.4)	89.9	224 (9.5)	10.1	2,228 (9.4)
Congestive heart failure	2,874 (13.4)	92.1	246 (10.4)	7.9	3,120 (13.1)
Peripheral vascular disease	5,464 (25.5)	88.6	704 (29.9)	11.4	6,168 (25.9)
Cerebrovascular disease	1,829 (8.5)	88.7	233 (9.9)	11.3	2,062 (8.7)
Chronic pulmonary disease	8,316 (38.8)	88.8	1,048 (44.5)	11.2	9,364 (39.4)
Rheumatologic disease	1,156 (5.4)	91.8	103 (4.4)	8.2	1,259 (5.3)
Peptic ulcer disease	418 (2)	94.8	23 (1)	5.2	441 (1.9)
Mild liver disease	1,662 (7.8)	91.3	158 (6.7)	8.7	1,820 (7.7)
Diabetes	5,147 (24)	91.4	486 (20.6)	8.6	5,633 (23.7)
Diabetes with complications	3,151 (14.7)	91.2	303 (12.9)	8.8	3,454 (14.5)
Hemiplegia or paraplegia	292 (1.4)	93	22 (0.9)	7	314 (1.3)
Renal disease	4,228 (19.7)	90.3	453 (19.2)	9.7	4,681 (19.7)
Moderate or severe liver disease	185 (0.9)	94.4	11 (0.5)	5.6	196 (0.8)

Data are presented No. (column %) row %, No. (column %), No., mean [SD], or as otherwise indicated.

## Results

**Figure 1:**
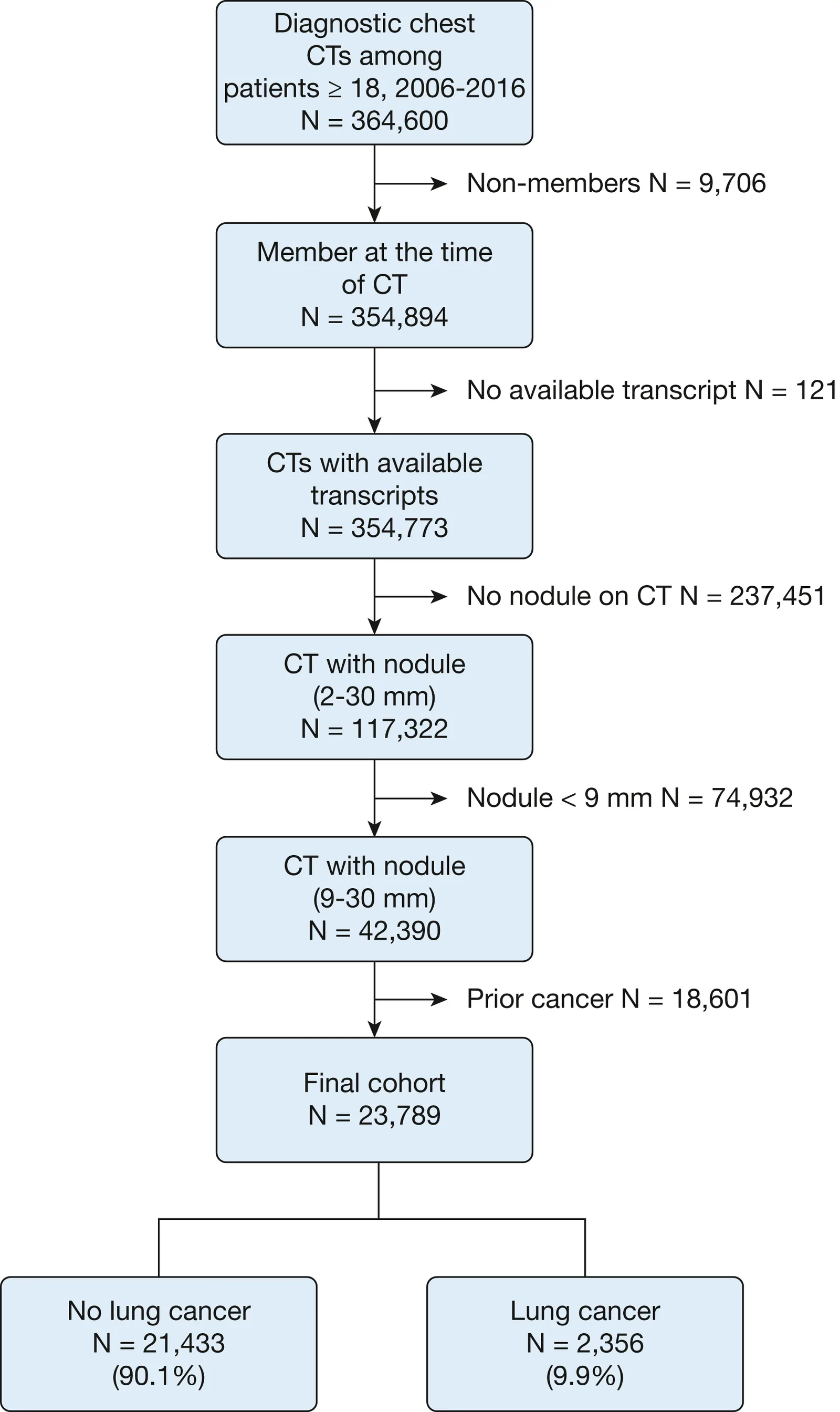
Cohort assembly.

From January 1, 2006, to December 31, 2016, there were 23,789 unique adult members with incidental lung nodules (9-30 mm in size) identified on chest CT scan (Fig 1). Cohort characteristics are shown in Table 1. Among them, 2,356 patients (9.9%) were diagnosed with lung cancer within 27 months of nodule identification. Nodule characteristics, including size, attenuation, lobe, and edge, were described for benign and cancerous nodules (Table 2). Overall, 63.3% of patients had imaging follow-up, including 60.1% of patients diagnosed with benign nodules and 92.9% of patients ultimately diagnosed with lung cancer (Table 3).

**Table 2:** Nodule Characteristics

Characteristic	No Lung Cancer (n = 21,433)		Lung Cancer (n = 2,356)		Total (N = 23,789)
Lobe					
Upper (including lingula, apex or apical)	7,858 (36.7)	87.2	1,158 (49.2)	12.8	9,016 (37.9)
Middle	1,832 (8.5)	90.8	186 (7.9)	9.2	2,018 (8.5)
Lower	7,532 (35.1)	91.8	671 (28.5)	8.2	8,203 (34.5)
> 1 lobe (if ≥ 2 nodules of same size present)	1,085 (5.1)	90.5	114 (4.8)	9.5	1,199 (5)
Not specified by radiologist	3,126 (14.6)	93.2	227 (9.6)	6.8	3,353 (14.1)
Nodule attenuation					
Solid	290 (1.4)	82.6	61 (2.6)	17.4	351 (1.5)
Part-solid	165 (0.8)	88.7	21 (0.9)	11.3	186 (0.8)
Nonsolid	1,489 (6.9)	92.9	113 (4.8)	7.1	1,602 (6.7)
Not specified by radiologist	19,489 (90.9)	90.0	2,161 (91.7)	10.0	21,650 (91)
Size of largest nodule, mm	15.7 [6.09]		19.5 [6.28]		16.1 [6.21]
Nodule size category, mm					
≥ 9 to 15	12,603 (58.8)	94.3	756 (32.1)	5.7	13,359 (56.2)
> 15 to 20	4,407 (20.6)	87.9	604 (25.6)	12.1	5,011 (21.1)
> 20 to 30	4,423 (20.6)	81.6	996 (42.3)	18.4	5419 (22.8)
Size of solid component (part-solid nodules), mm	16.5 [6.36]		22.6 [5.79]		17.2 [6.57]
Missing, n	21,268		2,335		23,603
Edge or margin					
Smooth	1,165 (5.4)	92.9	89 (3.8)	7.1	1,254 (5.3)
Lobulated	606 (2.8)	83.7	118 (5)	16.3	724 (3)
Irregular	3,213 (15)	90	358 (15.2)	10.0	3,571 (15)
Spiculated	1,287 (6)	67.2	628 (26.7)	32.8	1,915 (8)
Other	0 (0)	0	0 (0)	0	0 (0)
Not specified by radiologist	15,162 (70.7)	92.9	1,163 (49.4)	7.1	16,325 (68.6)

Data are presented as No. (column %) row %, No. (column %), or mean [SD].

**Table 3:** Follow-Up Diagnostic Tests, From Time of Index CT Scan to 27 Months

Diagnostic Test or Procedure	No Lung Cancer (n = 21,433)			Lung Cancer (n = 2,356)		
	No. of Individuals		No. of Events	No. of Individuals		No. of Events
	No.	%	No.	No.	%	No.
Imaging tests						
CT chest scan	12,197	56.9	17,854	1,954	82.9	3,635
PET scan or PET/CT scan	2,903	13.5	3,206	1,495	63.5	1,962
Either	12,883	60.1	21,060	2,188	92.9	5,597
Biopsy procedures						
Percutaneous lung biopsy	1,077	5.0	1,169	1,052	44.7	1,192
Bronchoscopy	864	4.0	996	396	16.8	478
Sampling of pleura or pleural fluid	433	2.0	566	259	11.0	385
Thoracoscopic surgery	218	1.0	218	717	30.4	739
Thoracotomy/open surgery	36	0.2	36	166	7.0	166
Mediastinotomy/mediastinoscopy	20	0.1	20	88	3.7	93
Cervical lymph node biopsy	165	0.8	178	121	5.1	127
Needle biopsy, not otherwise specified	226	1.1	258	74	3.1	77
Bone biopsy	88	0.4	96	60	2.5	64
Liver biopsy	160	0.7	174	42	1.8	45
Adrenal biopsy	62	0.3	65	11	0.5	11
Brain or spinal cord biopsy	9	0.0	9	5	0.2	6
Any	2,748	12.8	3,785	1,775	75.3	3,383

### Resource Utilization

When stratified by final diagnosis, patients with a lung cancer diagnosis had greater resource use across all imaging tests and biopsy procedures. Almost 93% of patients with lung cancer underwent imaging, including 82.9% who had at least 1 chest CT scan and 63.5% who underwent PET-CT imaging; 75.3% of patients with an eventual lung cancer diagnosis underwent invasive follow-up testing, including percutaneous biopsy (44.7%), bronchoscopy (16.8%), thoracoscopy (30.4%), and/or thoracotomy (7.0%). In contrast, 60.1% of patients with a benign nodule received additional imaging, and only 12.8% underwent invasive biopsy or surgical diagnosis (Table 3).

Resource utilization for imaging and biopsies was stratified by nodule size (9-15, 15-20, and 20-30 mm) and diagnosis (e-Table 1). Among patients with and without a lung cancer diagnosis, the overall likelihood of undergoing at least 1 follow-up evaluation did not differ substantially across nodule size categories. However, patterns of follow-up differed with nodule size. In particular, the rate of percutaneous biopsy was associated with increasing nodule size in both benign (3.2%, 6.9%, and 8.4%) and malignant nodules (40.7%, 45.5%, and 47.1%), respectively. A similar trend was observed for PET scan or PET-CT scan utilization in the malignant group (58.7%, 63.6%, and 67%), respectively. Although the frequency of surgical resections did not appear to vary by nodule size among patients with a lung cancer diagnosis, it increased from 0.9% for nodules measuring 9 to 15 mm to 1.8% for nodules measuring 20 to 30 mm among patients with a benign nodule. In total, 1,169 of 2,361 percutaneous biopsies (49.5%) and 996 of 1,474 bronchoscopies (67.6%) were performed in patients with benign nodules. A total of 1,159 thoracic surgical procedures (thoracoscopic or thoracotomy/open surgeries) were performed during follow-up, of which 254 (21.9%) occurred in patients with benign nodules (e-Table 1).

**Figure 2:**
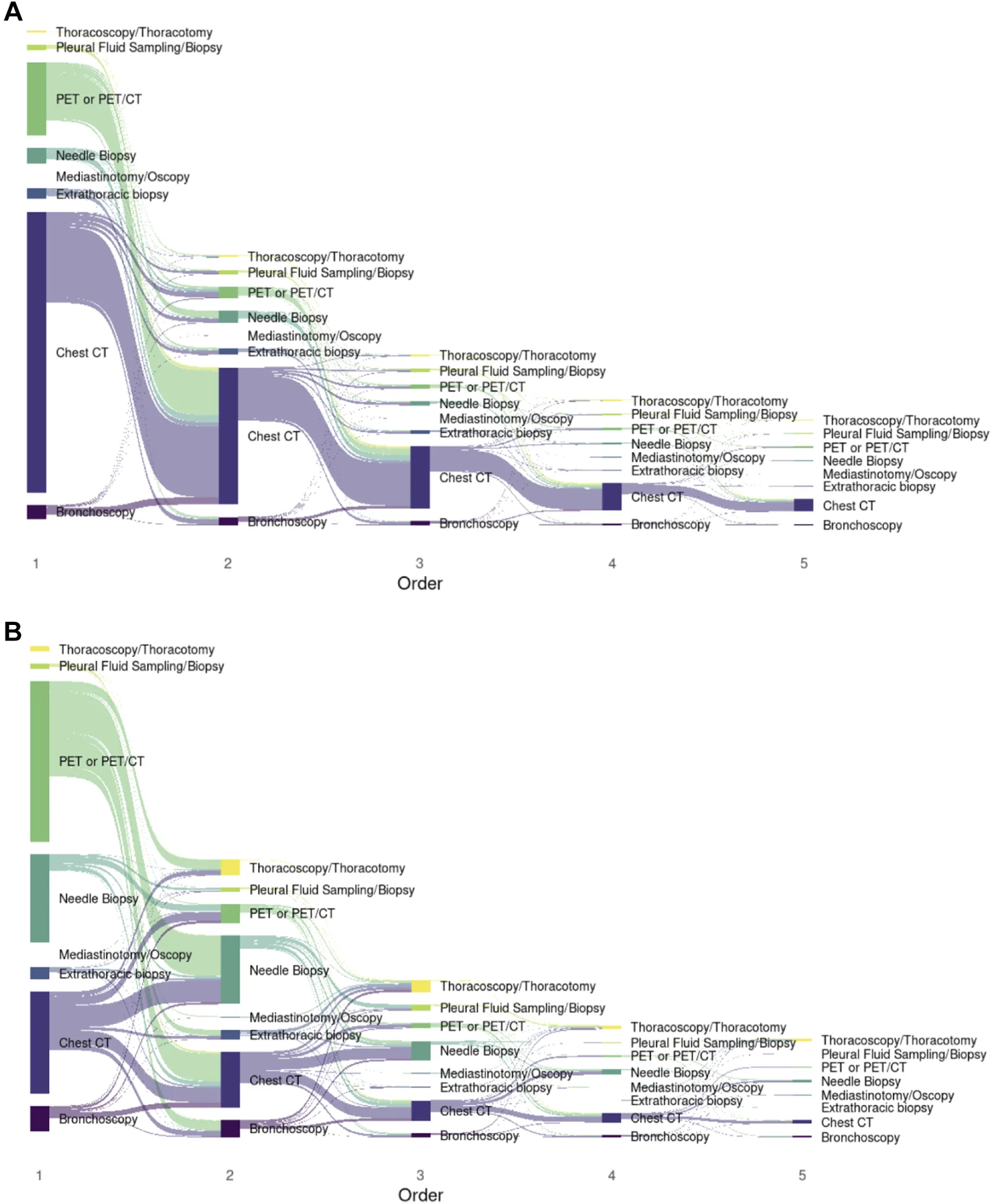
A, B, Sankey diagram including management pathways of (A) benign nodules and (B) malignant nodules.

Sankey plots showed highly variable management pathways with substantial differences between patients with benign vs malignant nodules (Fig 2). The predominant management approach for benign nodules involved follow-up chest CT scans, with relatively few invasive procedures performed. Patients with malignant nodules demonstrated more diverse management pathways, with relatively more PET scans and invasive procedures.

Within the Sankey plot of malignant lung nodules, distribution of invasive procedures was as follows. Within the first round, 642 unique patients underwent invasive procedures (34%), including 417 needle biopsies (22%), 118 bronchoscopies (6.3%), 58 extrathoracic biopsies (3%), 24 thoracoscopies/thoracotomies (1.3%), 24 pleural procedures (1.3%), and 1 mediastinotomy/mediastinoscopy (0.05%). Within the second round, 498 unique patients underwent invasive procedures (57%), including 325 needle biopsies (37%), 82 bronchoscopies (9.4%), 47 extrathoracic biopsies (5.3%), 79 thoracoscopies/thoracotomies (9%), 20 pleural procedures (2.2%), and 5 mediastinotomies/mediastinoscopies (0.5%). Within the third round, 219 unique patients underwent invasive procedures (62%) including 95 needle biopsies (27%), 19 bronchoscopies (5.4%), 8 extrathoracic biopsies (2.3%), 64 thoracoscopies/thoracotomies (18%), 29 pleural procedures (8.2%), and 4 mediastinotomies/mediastinoscopies (1.1%).

### Time to Diagnosis and Treatment

There were 1,079 patients with malignant nodules and available NDI information. Patients were stratified by NDI quartile with demographics recorded in e-Table 2. The median patient in the sample had an NDI of –0.1, indicating a level of deprivation like that of the Kaiser Permanente population at large. The median time to diagnosis across NDI quartiles was 40, 34, 45, and 39 days for quartiles 1 through 4, respectively (e-Table 3). The median time to treatment was 77, 81, 87, and 88 days across the same quartiles, respectively.

**Figure 3:**
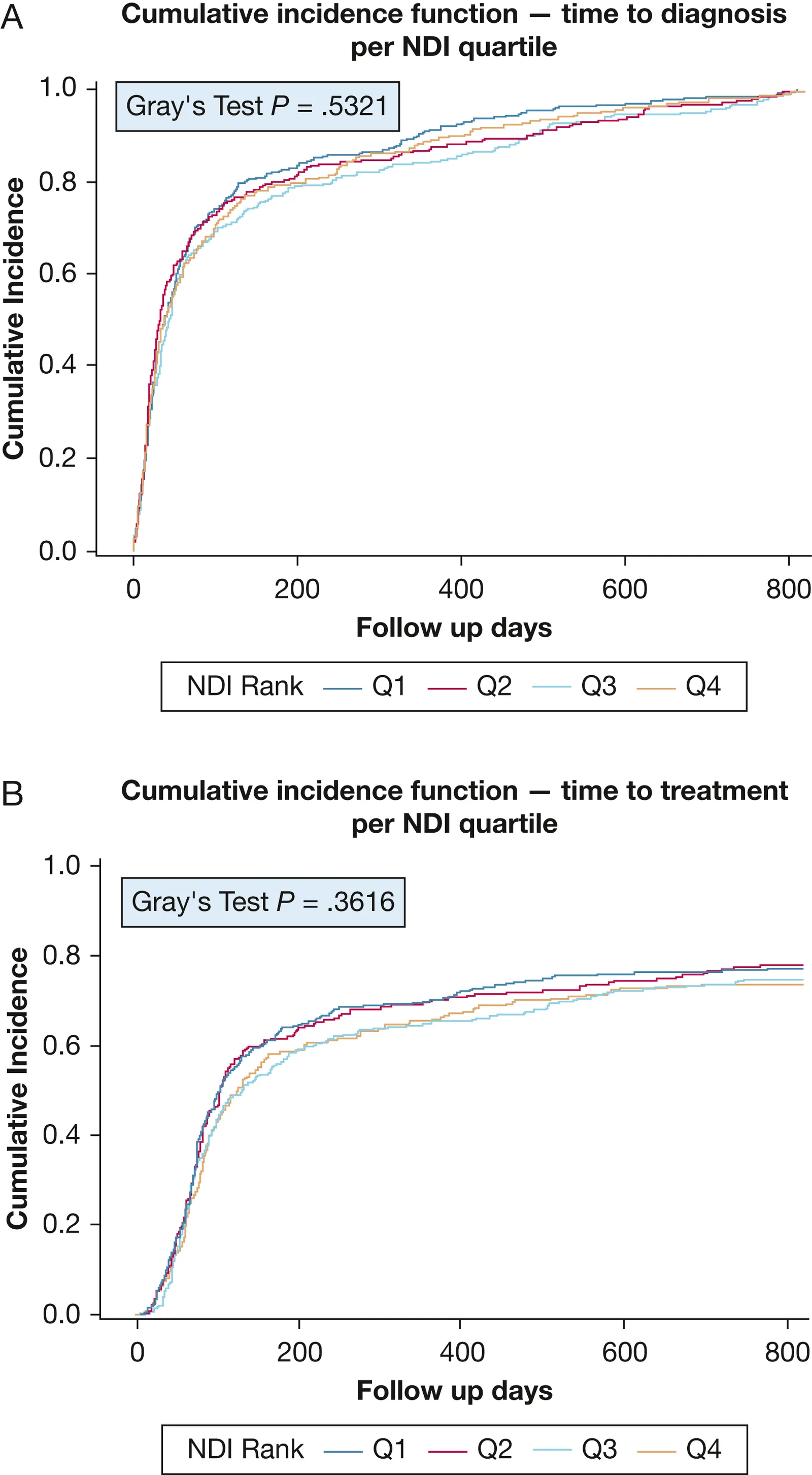
A, B, Cumulative incidence function including (A) time to diagnosis per NDI quartile and (B) time to treatment per NDI quartile. NDI = Neighborhood Deprivation Index; Q = quartile.

When analyzed as a categorical variable, there was no statistically significant association between NDI quartiles and time to diagnosis or time to treatment (e-Table 3, Fig 3). However, patients in the third quartile of deprivation were diagnosed approximately 29 days later than patients in the least deprived quartile (*P* = .06). For time to treatment, patients in the most deprived quartile were treated approximately 15 days later than patients in the least deprived quartile and were 18% less likely to be treated at a given time (hazard ratio, 0.82; 95% CI, 0.66-1.03; *P* = .08) (e-Table 3).

When analyzed as a continuous variable, there was no statistically significant relationship between the NDI and time to diagnosis (e-Table 3). However, there was a statistically significant relationship between the NDI and time to treatment (hazard ratio, 0.89; 95% CI, 0.81-0.97; *P* < .01), suggesting that for every SD increase in deprivation, patients were 11% less likely to receive treatment at any time.

## Discussion

In this study, we identified substantial differences in resource utilization between patients with benign and malignant nodules. As expected, patients diagnosed with lung cancer underwent more extensive imaging tests and invasive diagnostic procedures than those with benign nodules. Although a relatively small proportion of patients with benign nodules underwent invasive testing, the absolute number of invasive procedures performed on patients with benign nodules was substantial: 1 in 5 underwent surgical resection, 1 in 2 underwent percutaneous biopsy, and 2 in 3 bronchoscopies were performed in patients who ultimately did not have cancer.

Although some invasive evaluation among benign nodules is expected, given the uncertainty in pretest probability and overlapping radiographic features, these findings suggest potential overevaluation in a predominantly benign population. Although widely accepted benchmarks for benign biopsy rates do not exist, the Society of Thoracic Surgeons has recommended a benign surgical resection rate of < 10% in lung cancer screening populations.^11^ In this cohort, 21.9% of surgical resections were benign. In this context, we believe that a 10 to 15 percentage point reduction in invasive procedures performed in benign noduels represents a reasonable target for quality improvement programs.

At the same time, a striking proportion of patients received no follow-up imaging during the 27-month period. Overall, 36.7% of patients had no documented follow-up imaging, including 40.0% of patients with benign nodules and 7.1% of patients ultimately diagnosed with lung cancer. Although these patients with cancer may have appropriately proceeded directly to diagnostic tests, definitive treatment, or hospice care, the proportion of patients with benign nodules without follow-up imaging likely represents a substantial proportion of patients not receiving guideline-directed follow-up. This finding is consistent with prior studies reporting nearly one-half of patients with incidentally detected pulmonary nodules do not receive guideline-concordant care.^12^ Together, these findings highlight that there was too much care for some patients and too little care for others.

Sankey plots highlighted wide variability in patient management pathways. Follow-up CT scan was more prevalent among patients with benign nodules, whereas patients with malignant nodules underwent more complex pathways involving PET imaging and invasive procedures. This heterogeneity likely reflects differences in cancer risk and patient preferences, representing a clinical problem for which there may be warranted variation in practice.

### Timeliness of Diagnosis and Treatment

Analysis of timeliness in cancer diagnosis and treatment revealed a nuanced relationship with socioeconomic status. Overall, the median time to diagnosis across NDI quartiles did not differ significantly, ranging from 34 days in quartile 2 (shortest) to 45 days in quartile 3 (longest). These findings are comparable with prior work, which demonstrate a median time to diagnosis of approximately 1.3 months among patients receiving guideline-concordant care.^13^

In contrast, associations with socioeconomic deprivation emerged after diagnosis. Although NDI quartiles were not associated with time to diagnosis or treatment when analyzed categorically, patients in the third quartile of deprivation experienced an average 29-day delay in treatment compared with the least deprived quartile (e-Table 3). When analyzed as a continuous variable, a higher NDI score was significantly associated with treatment delay, with patients being 11% less likely to receive treatment at any given time for each SD increase in deprivation.

The absence of an association between deprivation and time to diagnosis suggests diagnostic evaluation proceeds at similar rates across socioeconomic classes, whereas barriers to initiating treatment—whether because of logistical challenges, health care access, or other social determinants—may become more pronounced after diagnosis.

Although direct comparison of rates is complicated by factors such as outcome definitions and baseline cancer risk, our findings reinforce concerns about overuse and variability in invasive testing. Like Wiener et al,^5^ we observed overevaluation in the benign nodule group, with 12.8% undergoing biopsy or surgery, and nearly one-half of all invasive procedures were ultimately performed in patients with benign nodules. This aligns with their report that 18% of patients were overevaluated and that 41.3% of those undergoing invasive procedures ultimately did not have cancer. Other real-world data (eg, an abstract from Thompson et al^14^) support this pattern.^14^^,^^15^

Beyond overevaluation, prior studies have demonstrated substantial gaps in guideline-concordant follow-up for incidentally detected pulmonary nodules. Work has shown that nearly one-half of patients do not receive guideline-concordant care, and socioeconomic disadvantage is associated with higher rates of inappropriate follow-up.^12^^,^^15^^,^^16^ Our findings complement this literature by characterizing the heterogeneity of diagnostic pathways and procedural intensity, and by demonstrating that socioeconomic deprivation is associated with differences in time to treatment despite similar time to diagnosis.

Efforts to improve consistency and reduce unnecessary interventions have included implementation of multidisciplinary nodule clinics and tumor boards, which promote guideline-concordant care while reducing provider bias and offering tailored management based on patient-specific needs.^17^ Resource availability for these approaches remains uneven across health systems.^18^ As a result, patients often receive care across a wide spectrum, including instances where more intensive diagnostic strategies lead to increased harm without a corresponding reduction in late-stage diagnoses.^13^ Our results add to this body of evidence and the need for more structured, nuanced approaches to nodule evaluation that balance diagnostic accuracy with patient safety and resource stewardship.^19^^,^^20^

### Limitations and Future Research

This study reflects a historical cohort evaluated between 2006 and 2016, which may predate widespread adoption of lung cancer screening and routine use of structured nodule management programs in some health systems. Patterns of care observed here may differ from contemporary practice. The introduction of multidisciplinary nodule programs likely standardized follow-up, thereby reducing variation and overuse of invasive procedures. At the same time, incidental nodules detected outside of screening programs remain common, and variation in management persists even in modern practice.

Although this was a cohort study with longitudinal follow-up, causal inference is limited because patients were not randomly assigned to NDI categories. As a result, the observational design limits our ability to establish causal relationships and leaves the possibility of unmeasured confounding despite statistical adjustments. Possible sources of unmeasured confounding include patient preferences, physician practice styles, and usage of tumor boards or multidisciplinary clinics. In addition, although nodule characteristics (eg, size, attenuation) were included in the regression model as plausible confounders, missing specification of a large proportion of nodule characteristics by radiologists may have limited our ability to sufficiently adjust for how these factors affect model outcomes.

The study population was drawn exclusively from Kaiser Permanente Southern California, which may limit the generalizability of our findings to other populations. Kaiser members typically have integrated care with consistent access to health care services. This may not reflect the experiences of individuals in other systems or those without insurance. A broader patient population may show wider variation in resource utilization, different management biases, and/or more pronounced disparities in time to diagnosis and treatment because of decreased standardization across health centers.

Finally, these findings are not generalizable to smaller nodules, which comprise most incidental nodules and are typically managed by chest CT scan follow-up. Furthermore, although the NDI is a robust measure of socioeconomic status, it may not fully capture other factors such as health literacy, social support, or cultural barriers.

## Interpretation

In this study, we describe wide variation in resource utilization and care practices for patients with both benign and malignant lung nodules measuring ≥ 9 mm in size after incidental detection on chest CT scan. In addition, although neighborhood socioeconomic deprivation demonstrated no statistically significant relationship with timeliness to diagnosis, deprivation was associated with longer time to treatment.

## Funding/Support

The authors have reported to *CHEST Pulmonary* that no funding was received for this study.

## Financial/Nonfinancial Disclosures

None declared.

## Supplementary Data

e-Online Data
