# Supplementary material for: Resource Utilization and Timeliness of Care in Patients With Incidentally Detected Lung Nodules
Source: CHEST Pulm. 2026 Jul 3;4(3):100270. doi: 10.1016/j.chpulm.2026.100270 (PMC13571422; doi:10.1016/j.chpulm.2026.100270)
Supplement: Supplementary file 1 [file mmc1.docx]

**e-Table 1.** Resource utilization by nodule size and final diagnosis

| **Measure** | **Nodule Size (mm)** | **No Lung Cancer** | **Lung Cancer** | **Overall** |
| --- | --- | --- | --- | --- |
| **Patients with ≥1 chest CT scan, n (%)** | 9 to 15 | 7347 (58.3) | 645 (85.3) | 7992 (59.8) |
| **col pct** | >15 to 20 | 2420 (54.9) | 505 (83.6) | 2925 (58.6) |
|  | >20 to 30 | 2430 (54.9) | 804 (80.7) | 3234 (59.7) |
|  | Any Size | 12197 (56.9) | 1954 (82.9) | 14151 (59.5) |
| **Total number of chest CT scans, n** | 9 to 15 | 10665 | 1157 | 11822 |
|  | >15 to 20 | 3518 | 933 | 4451 |
|  | >20 to 30 | 3671 | 1545 | 5216 |
|  | Any Size | 17854 | 3635 | 21489 |
| **Patients with ≥1 PET or PET/CT scan, n (%)** | 9 to 15 | 1625 (12.9) | 444 (58.7) | 2069 (15.5) |
|  | >15 to 20 | 656 (14.9) | 384 (63.6) | 1040 (20.8) |
|  | >20 to 30 | 622 (14.1) | 667 (67) | 1289 (23.8) |
|  | Any Size | 2903 (13.5) | 1495 (63.5) | 4398 (18.5) |
| **Total number of PET or PET/CT scans, n** | 9 to 15 | 1782 | 578 | 2360 |
|  | >15 to 20 | 722 | 489 | 1211 |
|  | >20 to 30 | 702 | 895 | 1597 |
|  | Any Size | 3206 | 1962 | 5168 |
| **Patients with ≥1 percutaneous needle biopsy, n (%)** | 9 to 15 | 403 (3.2) | 308 (40.7) | 711 (5.3) |
|  | >15 to 20 | 303 (6.9) | 275 (45.5) | 578 (11.5) |
|  | >20 to 30 | 371 (8.4) | 469 (47.1) | 840 (15.5) |
|  | Any Size | 1077 (5.0) | 1052 (44.7) | 2129 (9.0) |
| **Total number of percutaneous needle biopsies, n** | 9 to 15 | 434 | 355 | 789 |
|  | >15 to 20 | 326 | 308 | 634 |
|  | >20 to 30 | 409 | 529 | 938 |
|  | Any Size | 1169 | 1192 | 2361 |
| **Patients with ≥1 bronchoscopy, n (%)** | 9 to 15 | 447 (3.5) | 133 (17.6) | 580 (4.3) |
|  | >15 to 20 | 181 (4.1) | 96 (15.9) | 277 (5.5) |
|  | >20 to 30 | 236 (5.3) | 167 (16.8) | 403 (7.4) |
|  | Any Size | 864 (4.0) | 396 (16.8) | 1260 (5.3) |
| **Total number of bronchoscopies, n** | 9 to 15 | 526 | 162 | 688 |
|  | >15 to 20 | 207 | 111 | 318 |
|  | >20 to 30 | 263 | 205 | 468 |
|  | Any Size | 996 | 478 | 1474 |
| **Patients with ≥1 extrapulmonary biopsy, n (%)** | 9 to 15 | 586 (4.6) | 152 (20.1) | 738 (5.5) |
|  | >15 to 20 | 217 (4.9) | 119 (19.7) | 336 (6.7) |
|  | >20 to 30 | 251 (5.7) | 242 (24.3) | 493 (9.1) |
|  | Any Size | 1054 (4.9) | 513 (21.8) | 1567 (6.6) |
| **Total number of extrapulmonary biopsies, n** | 9 to 15 | 719 | 221 | 940 |
|  | >15 to 20 | 285 | 157 | 442 |
|  | >20 to 30 | 333 | 331 | 664 |
|  | Any Size | 1337 | 709 | 2046 |
| **Patients with ≥1 surgical procedure, n (%)** | 9 to 15 | 110 (0.9) | 258 (34.1) | 368 (2.8) |
|  | >15 to 20 | 56 (1.3) | 236 (39.1) | 292 (5.8) |
|  | >20 to 30 | 78 (1.8) | 373 (37.4) | 451 (8.3) |
|  | Any Size | 244 (1.1) | 867 (36.8) | 1111 (4.7) |
| **Total number of surgical procedure, n** | 9 to 15 | 115 | 273 | 388 |
|  | >15 to 20 | 59 | 244 | 303 |
|  | >20t o 30 | 80 | 388 | 468 |
|  | Any Size | 254 | 905 | 1159 |

**e-Table 2.** Demographic information of patients with a lung cancer diagnosis within 27 months, by NDI quartile

| **Parameter** | **NDI Q1** | **NDI Q2** | **NDI Q3** | **NDI Q4** | | **Overall** |
| --- | --- | --- | --- | --- | --- | --- |
| NDI^*^ (SD) | -0.8 (0.19) | -0.4 (0.15) | 0.3 (0.24) | 1.5 (0.55) | | 0.1 (0.9) |
| Age |  |  |  |  | |  |
| N | 292 | 257 | 291 | 239 | | 1079 |
| Mean (SD) | 70.1 (10.52) | 70.1 (9.98) | 70.1 (10.48) | 69.7 (10.13) | | 70.0 (10.28) |
| Median | 71 | 71 | 71 | 70 | | 71 |
| Q1, Q3 | 63.0, 78.0 | 65.0, 77.0 | 63.0, 77.0 | 64.0, 77.0 | | 64.0, 77.0 |
| Range | (24.0-94.0) | (43.0-92.0) | (28.0-92.0) | (36.0-92.0) | | (24.0-94.0) |
|  |  |  |  |  | |  |
| Gender |  |  |  |  | |  |
| Male | 142 (48.6%) | 100 (38.9%) | 132 (45.4%) | 98 (41%) | | 472 (43.7%) |
| Female | 150 (51.4%) | 157 (61.1%) | 159 (54.6%) | 141 (59%) | | 607 (56.3%) |
|  |  |  |  |  | |  |
| Race/Ethnicity |  |  |  |  | |  |
| White | 209 (71.6%) | 191 (74.3%) | 175 (60.1%) | 91 (38.1%) | | 666 (61.7%) |
| Asian | 14 (4.8%) | 10 (3.9%) | 28 (9.6%) | 60 (25.1%) | | 112 (10.4%) |
| Black | 25 (8.6%) | 25 (9.7%) | 61 (21%) | 61 (25.5%) | | 172 (15.9%) |
| Hispanic | 42 (14.4%) | 28 (10.9%) | 26 (8.9%) | 26 (10.9%) | | 122 (11.3%) |
| Other/Unknown | 2 (0.7%) | 3 (1.2%) | 1 (0.3%) | 1 (0.4%) | | 7 (0.6%) |
|  |  |  |  |  | |  |
| Insurance |  |  |  |  | |  |
| Medicare/Medicaid | 194 (66.4%) | 178 (69.3%) | 194 (66.7%) | 163 (68.2%) | | 729 (67.6%) |
| Commercial/Private Pay | 98 (33.6%) | 79 (30.7%) | 97 (33.3%) | 76 (31.8%) | | 350 (32.4%) |
|  |  |  |  |  | |  |
| Smoking status |  |  |  |  | |  |
| Missing/Never/Passive Smoker | 90 (30.8%) | 56 (21.8%) | 73 (25.1%) | 58 (24.3%) | | 277 (25.7%) |
| Current/former smoker | 202 (69.2%) | 201 (78.2%) | 218 (74.9%) | 181 (75.7%) | | 802 (74.3%) |
|  |  |  |  |  | |  |
| BMI |  |  |  |  | |  |
| Normal | 118 (40.4%) | 92 (35.8%) | 105 (36.1%) | 76 (31.8%) | | 391 (36.2%) |
| Underweight | 6 (2.1%) | 10 (3.9%) | 16 (5.5%) | 11 (4.6%) | | 43 (4%) |
| Overweight | 108 (37%) | 87 (33.9%) | 91 (31.3%) | 87 (36.4%) | | 373 (34.6%) |
| Obese | 60 (20.5%) | 68 (26.5%) | 79 (27.1%) | 65 (27.2%) | | 272 (25.2%) |
|  |  |  |  |  |  | |
| Weighted Charlson Score |  |  |  |  | |  |
| 0 | 75 (25.7%) | 66 (25.7%) | 50 (17.2%) | 45 (18.8%) | | 236 (21.9%) |
| 1 | 81 (27.7%) | 59 (23%) | 67 (23%) | 59 (24.7%) | | 266 (24.7%) |
| 2 | 59 (20.2%) | 48 (18.7%) | 67 (23%) | 46 (19.2%) | | 220 (20.4%) |
| 3+ | 77 (26.4%) | 84 (32.7%) | 107 (36.8%) | 89 (37.2%) | | 357 (33.1%) |
|  |  |  |  |  | |  |
| Chronic pulmonary disease |  |  |  |  | |  |
| No | 190 (65.1%) | 141 (54.9%) | 159 (54.6%) | 134 (56.1%) | | 624 (57.8%) |
| Yes | 102 (34.9%) | 116 (45.1%) | 132 (45.4%) | 105 (43.9%) | | 455 (42.2%) |
|  |  |  |  |  | |  |
| Congestive heart failure |  |  |  |  | |  |
| No | 266 (91.1%) | 238 (92.6%) | 265 (91.1%) | 216 (90.4%) | | 985 (91.3%) |
| Yes | 26 (8.9%) | 19 (7.4%) | 26 (8.9%) | 23 (9.6%) | | 94 (8.7%) |
|  |  |  |  |  | |  |
| Renal disease |  |  |  |  | |  |
| No | 231 (79.1%) | 207 (80.5%) | 233 (80.1%) | 187 (78.2%) | | 858 (79.5%) |
| Yes | 61 (20.9%) | 50 (19.5%) | 58 (19.9%) | 52 (21.8%) | | 221 (20.5%) |
|  |  |  |  |  | |  |
| Nodule Size |  |  |  |  | |  |
| >8 to 15 | 78 (26.7%) | 87 (33.9%) | 89 (30.6%) | 68 (28.5%) | | 322 (29.8%) |
| >15 | 214 (73.3%) | 170 (66.1%) | 202 (69.4%) | 171 (71.5%) | | 757 (70.2%) |
|  |  |  |  |  | |  |
| Nodule Edge |  |  |  |  | |  |
| All Others | 166 (56.8%) | 150 (58.4%) | 174 (59.8%) | 148 (61.9%) | | 638 (59.1%) |
| Irregular or Spiculated | 126 (43.2%) | 107 (41.6%) | 117 (40.2%) | 91 (38.1%) | | 441 (40.9%) |
|  |  |  |  |  | |  |

*NDI = Neighborhood deprivation index

**e-Table 3.** Associations between NDI and time to diagnosis and treatment as categorical and continuous variables

| **Time to diagnosis (categorical)** | Median (IQR) | Unadjusted difference, days | Adjusted difference, days | Pr > \|t\| |
| --- | --- | --- | --- | --- |
| NDI Q1 | 40 (20-110) | Referent | Referent |  |
| NDI Q2 | 34 (18-110) | 16.3 | 6.2 | 0.69 |
| NDI Q3 | 45 (19-154) | 33.3 | 28.9 | 0.06 |
| NDI Q4 | 39 (19-128) | 14.9 | 17.5 | 0.31 |
| **Time to diagnosis (continuous)** | - | - | 6.8 | 0.32 |
| **Time to treatment (categorical)** |  |  | Adjusted HR | Pr > Chisq |
| NDI Q1 | 77 (57, 133) | Referent | Referent |  |
| NDI Q2 | 81 (55, 131) | 13 | 1.02 (0.84 – 1.24) | 0.86 |
| NDI Q3 | 87 (58, 172) | 29 | 0.92 (0.72-1.12) | 0.42 |
| NDI Q4 | 88 (61, 151) | 15 | 0.82 (0.66-1.03) | 0.08 |
| **Time to treatment (continuous)** | - | - | 0.89 (0.81 – 0.97) | > 0.01 |

^*^Models adjusted for nodule characteristics (size, attenuation, edge), patient characteristics (race, age, gender, insurance type), comorbidities (smoking history, BMI, Charlson comorbidity index, COPD, CHF, renal disease), and medical center where care was received
